# Supplementary material for: Synaptic Plasticity-Enhancing and Cognitive-Improving Effects of Standardized Ethanol Extract of Perilla frutescens var. acuta in a Scopolamine-Induced Mouse Model
Source: Int J Mol Sci. 2025 Oct 12;26(20):9925. doi: 10.3390/ijms26209925 (PMC12562524; doi:10.3390/ijms26209925)
Supplement: Supplementary file 1 [file ijms-26-09925-s001.zip › ijms-3852472-supplementary.pdf]

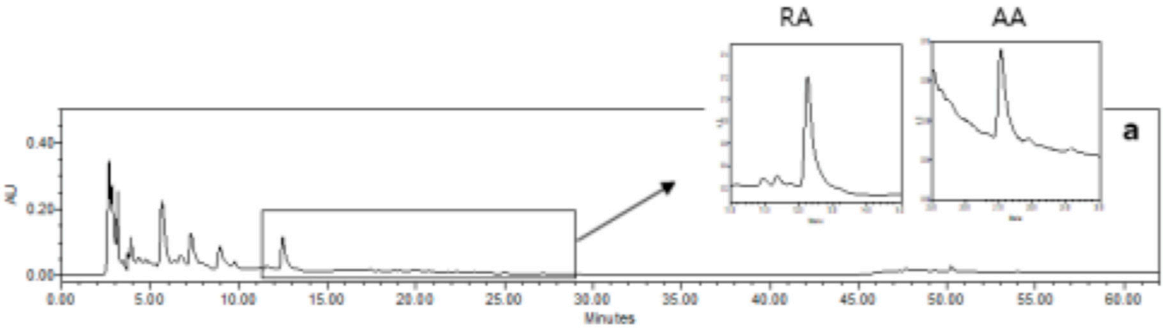

Supplemental Figure S1. HPLC chart for rosmarininc acid in PE. RA, rosmarinic acid. AA, alpha-asarone.

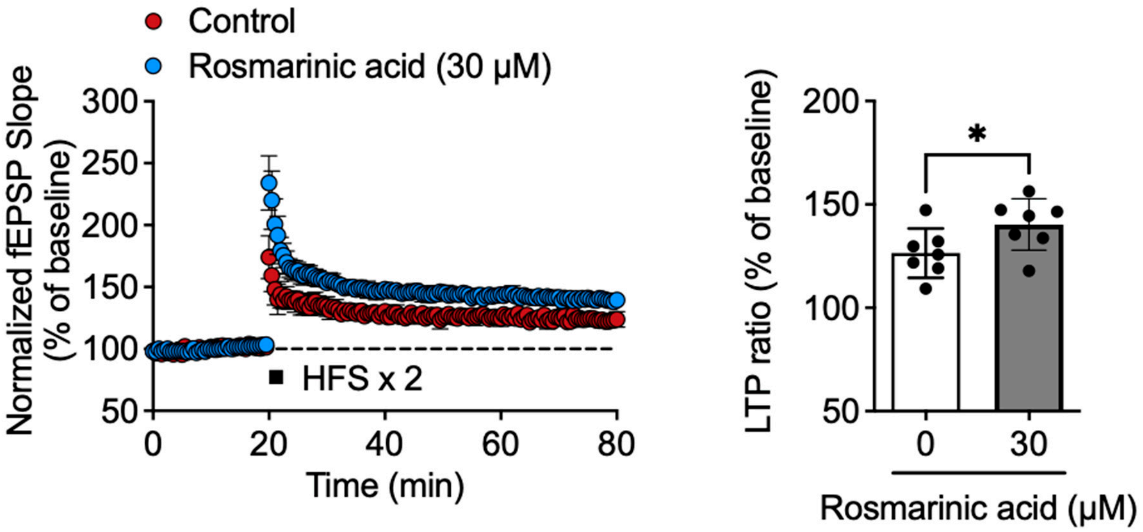

Supplemental Figure S2. Effect of rosmarinic acid on hippocampal LTP.
